# Evaluating the Use of Mobile Health Technology in Older Adults With Heart Failure: Mixed-Methods Study

**DOI:** 10.2196/12178

**Published:** 2018-12-04

**Authors:** Leanne L Lefler, Sarah J Rhoads, Melodee Harris, Ashley E Funderburg, Sandra A Lubin, Isis D Martel, Jennifer L Faulkner, Janet L Rooker, Deborah K Bell, Heather Marshall, Claudia J Beverly

**Affiliations:** 1 College of Nursing University of Arkansas for Medical Sciences Little Rock, AR United States; 2 College of Nursing University of Tennessee Health Science Center Memphis, TN United States; 3 Department of Family and Preventive Medicine University of Arkansas for Medical Sciences Fayetteville, AR United States; 4 Department of Family and Preventive Medicine University of Arkansas for Medical Sciences Little Rock, AR United States; 5 Department of Cardiology University of Arkansas for Medical Science Little Rock, AR United States

**Keywords:** heart failure, remote monitoring, mHealth, older adults, feasibility, self-management

## Abstract

**Background:**

Heart failure (HF) is associated with high rates of hospitalizations, morbidity, mortality, and costs. Remote patient monitoring (mobile health, mHealth) shows promise in improving self-care and HF management, thus increasing quality of care while reducing hospitalizations and costs; however, limited information exists regarding perceptions of older adults with HF about mHealth use.

**Objective:**

This study aimed to compare perspectives of older adults with HF who were randomized to either (1) mHealth equipment connected to a 24-hour call center, (2) digital home equipment, or (3) standard care, with regard to ease and satisfaction with equipment, provider communication and engagement, and ability to self-monitor and manage their disease.

**Methods:**

We performed a pilot study using a mixed-methods descriptive design with pre- and postsurveys, following participants for 12 weeks. We augmented these data with semistructured qualitative interviews to learn more about feasibility, satisfaction, communication, and self-management.

**Results:**

We enrolled 28 patients with HF aged 55 years and above, with 57% (16/28) male, 79% (22/28) non-Hispanic white, and with multiple comorbid conditions. At baseline, 50% (14/28) rated their health fair or poor and 36% (10/28) and 25% (7/28) were very often/always frustrated and discouraged by their health. At baseline, 46% (13/28) did not monitor their weight, 29% (8/28) did not monitor their blood pressure, and 68% (19/28) did not monitor for symptoms. Post intervention, 100% of the equipment groups home monitored daily. For technology anxiety, 36% (10/28) indicated technology made them nervous, and 32% (9/28) reported fear of technology, without significant changes post intervention. Technology usability post intervention scored high (91/100), reflecting ease of use. A majority indicated that a health care provider should be managing their health, and 71% reported that one should trust and not question the provider. Moreover, 57% (16/28) believed it was better to seek professional help than caring for oneself. Post intervention, mHealth users relied more on themselves, which was not mirrored in the home equipment or standard care groups. Participants were satisfied with communication and engagement with providers, yet many described access problems. Distressing symptoms were unpredictable and prevailed over the 12 weeks with 79 provider visits and 7 visits to emergency departments. The nurse call center received 872 readings, and we completed 289 telephone calls with participants. Narrative data revealed the following main themes: (1) traditional communication and engagement with providers prevailed, delaying access to care; (2) home monitoring with technology was described as useful, and mHealth users felt secure knowing that someone was observing them; (3) equipment groups felt more confident in self-monitoring and managing; and finally, (4) uncertainty and frustration with persistent health problems.

**Conclusions:**

mHealth equipment is feasible with potential to improve patient-centered outcomes and increase self-management in older adults with HF.

## Introduction

### Background

Heart failure (HF) is a major health problem worldwide. HF accounts for approximately 20% of all hospital discharges in older adults—6.5 million people in the United States—and is increasing significantly as adults are living longer [1]. It is also associated with high symptom burden, comorbidity, and mortality [2-4]. Adults with HF challenge current health care systems because of the complexity of the disease, need for continuous management, coordination of care with multiple providers, and the need to support patients in community settings. Proactive symptom detection combined with earlier health care intervention provides a greater chance in reduction of poor outcomes than reactive treatment where patients wait until they have a serious symptom such as angina or dyspnea [3,4].

Self-care is the cornerstone to HF treatment [5], especially as related to home monitoring for subclinical congestion [6]; however, many patients do not understand how or what to self-monitor [7]. It is important for patients to implement ongoing assessment strategies to identify early indications of impending exacerbations through self-monitoring of weight, blood pressure (BP), and symptoms. Physiological changes that may indicate subclinical congestion may be monitored through a range of strategies ranging from home observation to routine monitoring of vital signs (such as BP and heart rate) to auto monitoring through implantable devices [6]. Patients with HF can prevent comorbidity, premature mortality, and costly hospitalizations through early recognition of decompensation and self-care strategies at home [8-10].

Effective communication with a care team is essential in the multifaceted management of patients with HF [5]. Systematic reviews and meta-analyses support that the care team is a primary characteristic associated with reduced morbidity and mortality [11], and patients who have improved communication with their health care team are known to have increased adherence to the treatment plan [12] and have increased satisfaction [13].

The increased utilization of remote monitoring, using connected mobile health (mHealth) devices at home, is one way to increase self-management and communication. The Center for Connected Health Policy defines remote patient monitoring as “the use of digital technologies to collect medical and other forms of health data that electronically transmits information securely to health care providers” [14]. Remote patient monitoring using mHealth equipment allows in-home monitoring of patient vital signs, such as BP and weight, the same as monitoring in the exam room, allowing clinicians to monitor patients from almost anywhere. Studies using mHealth systems provide evidence that mobile monitoring reduces morbidity [2,3,15,16] and mortality [2]; however, a systematic review reports inconsistent outcomes [17]. In addition to improving morbidity and mortality, studies found fewer emergency department (ED) visits and hospitalizations with remote monitoring [16,18,19]. Despite a growing body of research demonstrating positive physiological outcomes and lower health care costs, few studies have considered the patient’s perspective on the potential benefits and burdens associated with the in-home monitoring of HF. In addition, inconsistent findings in the literature are likely related to variations in mHealth monitoring strategies [2-4,15,20]. This inconsistency demonstrates the need for more focused research in comparing approaches with the monitoring of patients with HF to determine which methods are most effective in improving overall patient outcomes, especially in older adults.

### Aims

The purposes of this pilot study were (1) to examine the feasibility of using mHealth equipment with community- dwelling older adults who suffer from HF and (2) to examine their perspectives as they used mHealth equipment compared with in-home equipment and standard care, specifically patient ease and satisfaction with using the equipment, health care team communication and engagement, and ability to self-monitor and manage their disease.

## Methods

### Design

This was a mixed-methods study using a descriptive explanatory design. We used survey methodology along with semistructured qualitative interviews to obtain a better understanding of patient experiences. The study was approved by the university institutional review board.

### Sampling and Recruitment

Participants were recruited from 2 cardiology clinics in a southern state. Provider’s office personnel identified potential participants who were interested in participating. A research assistant telephoned the participants and conducted a brief screening of the inclusion and exclusion criteria (see Textbox 1). If eligible, the research assistant scheduled an appointment to meet with the patient, and the caregiver(s) were also encouraged to attend. Participants were provided study information for fully informed consent.

**Research participants’ inclusion and exclusion criteria.**

**Inclusion criteria**

- Aged 55 years and above
- Current diagnosis of heart failure
- Successfully completes capacity to consent given during initial screening process
- At least 2 weeks of no hospitalizations before enrollment
- Able to stand to take daily weight measurements and read values independently or with the assistance of a caregiver
- Has working telephone reachable via text or call 24 hours/7 days a week
- English as primary language

**Exclusion criteria**

- Involved in other studies
- Involved in hospital case management
- Living in nursing home setting
- Diagnosis of dementia as indicated by a St Louis University Mental Status (SLUMS) score <20

#### Data Collection Randomization to Groups

After baseline measures were taken, we randomized enrolled participants into 3 groups using statistician-generated random allocation cards that were sealed in envelopes: (1) mHealth equipment group, (2) in-home equipment group, and (3) standard care group. Each group received a different self-management intervention.

#### Self-Management Interventions

Subjects in the mHealth group received a Cloud DX Connected Health Kit containing an Android Health Tablet with Bluetooth-paired body weight scale and the Pulsewave Universal Serial Bus BP wrist monitor. Subjects took daily BP and weight readings using the mHealth Cloud DX equipment. The equipment allowed real-time collection and monitoring of patients’ values, instantly accessible 24 hours a day, 7 days a week by both patients and their clinicians, via a wireless gateway that transmitted results to a secure cloud-based clinician portal. The participants’ daily weight and BP readings were remotely sent to the hospital’s call center where we employed registered nurses specially trained to triage these patients using physician-study team–developed protocols. These protocols standardized the triage process, ensuring consistency in patient management.

Data collected from the call center included number of calls made by triage nurse staff, the patients, and the nature and outcomes of these calls. Readings falling outside predetermined parameters triggered notifications, alerting research staff and call center nurses. After receiving an alert, a call center nurse then contacted the patient and began the triage process based on alert type. For BP readings falling above or below set parameters, the call center nurse first directed the patient to relax for 15 min, then to retake their BP. If the second reading was in the normal range, no further action was required. If the reading remained outside of the parameters, the nurse asked a series of questions to determine patient needs. The nurse asked questions about physical symptoms (chest pain, shortness of breath, and activity level), mental symptoms (stress levels and sleeping habits), medications (when BP readings or new medications were last taken), and dietary questions (does patient follow a low sodium diet and when did the patient last eat). An alert triggered by weight gain required additional questions about presence of edema, diuretics used, and fluid intake. This combination of questions and responses determined the next steps in triaging, whether it was to take medications and recheck BP in an hour, contacting the patients’ physician’s office for orders, or a recommendation to go to the local ED. Daily readings not received by 1 pm resulted in a reminder call from the call center. We used the compiled intervention data to record patient compliance, number and types of incidents requiring triage, adherence to triage protocols, and intervention outcomes.

The in-home equipment group received a standard Medline Plus digital BP wrist monitor, an Escali digital weight scale. Neither of these devices were connected to the call center or the software system. They were instructed to use this equipment and to record their daily BP and weight readings via pencil and paper using a log or diary that we supplied. They were also instructed of the parameters their provider specified for their BP and weight readings and to call their provider if readings were out of range.

The standard of care (SoC) group did not receive any equipment and were asked to continue following regular care instructions provided by their health care team and current self-monitoring routine. We did not encourage nor discourage the SoC subjects to change their daily self-management.

### Survey Data and Instruments

A total of 3 surveys were used in collecting participant data: (1) baseline survey, (2) postintervention survey, and (3) weekly symptom and status phone survey. The baseline survey captured demographic data and participant perceptions across 6 health domains: (1) general health and symptoms, (2) self-care perceptions, (3) provider care perceptions, (4) communication and engagement with health care team, (5) monitoring adherence, and (6) technology anxiety. See Table 1 for a description of the questionnaires adapted for use in the survey. The survey was administered in person by a research team member. All responses were entered directly into LimeSurvey, a Web-based service platform.

All participants received a weekly phone call over the course of the 12-week study to follow events prospectively. The weekly symptom and status survey for the 2 equipment groups consisted of 22 questions across the following 6 domains: (1) current symptoms; (2) equipment status/issues; (3) medical visits/emergencies; (4) symptom changes; (5) adherence behavior for medication, diet, and exercise; and (6) BP and weight daily log adherence (see Table 2). The SoC group received a phone call to mimic attention but did not receive the survey; they were instead asked whether their health condition caused them to seek medical care (doctor or ED visit).

**Table 1:** Baseline/postsurvey questionnaire instruments.

Survey domain and instruments		Items, n	Measures
**General health and symptoms**			
	Self-Rated Health Scale [21]	1	Patient’s self-rated overall health
	Health Distress Scale [22]	4	Distress levels triggered by current health problems/symptoms
**Self-care perceptions**			
	Psychological Empowerment Scale [23], adapted	4	Importance of self-care, self-efficacy, decision-making abilities, and control over own health and health outcomes
**Provider care perceptions**			
	Krantz Health Opinion Survey [24], adapted	5	Patient perceptions regarding provider care including active involvement in self-treatment and information seeking with regards to staying informed and a part of medical decisions
**Patient communication and engagement**			
	Medicare Fee-for-Service Consumer Assessment of Health Plans Survey [25], adapted	4	Patient satisfaction with health plan, medical care, and overall communication with health care team
**Monitoring adherence**			
	Morisky et al Medication Adherence Scale [26], adapted	3	Patient fidelity to self-monitoring upon receiving information from their health care provider on how to monitor signs and symptoms of heart failure
**Technology anxiety**			
	Technology Acceptance Model [27], adapted	2	Patient anxiety/stress levels when working with technology
**Equipment usability and self-management (post survey only)**			
	System Usability Scale [28]	10	Effectiveness, efficiency, and user satisfaction when using a system or piece of technology
	Feasibility (author derived)	1	Feasibility of using equipment daily to monitor symptoms at home

**Table 2:** Weekly symptom and status survey.

Survey		Items, n	Measures
**Symptom Status Questionnaire-Heart Failure [29]**		7	Presence of current physical heart failure symptoms
	Frequency	1	Frequency of symptom occurrence
	Severity	1	Severity of symptoms
	Distress levels	1	Extent of symptom-induced stress
Equipment status/issues		2	Equipment problems and troubleshooting
Doctor/emergency department visits		2	Health-related clinic and/or emergency department visits; reason for visits
Lifestyle behaviors		5	General questions about sleep, daily activities, diet, exercise, and medications
Symptom improvement		2	Improvement in current symptoms and/or newly occurring symptoms
BP^a^/weight log adherence		2	Daily self-recording of BP and weight—home equipment group only
BP/weight changes		2	Notable changes in BP or weight readings—home equipment group only

^a^BP: blood pressure.

Finally, the postintervention survey was identical to the baseline survey except for removal of the demographic questions and addition of an equipment usability and self-monitoring questionnaire consisting of 11 questions. As the SoC group did not receive any equipment, they did not receive these additional questions. The postintervention survey was administered via telephone at 12-weeks in the same manner.

### Qualitative Methods

The purpose of the qualitative approach was to learn more about feasibility, satisfaction, communication, and self-management implications of using mHealth technology compared with in-home equipment and standard care in older adults with HF. A content analysis was used as the qualitative interpretive guide for these narratives according to criterion published by Boreus and Bergstrom [30]. This strategy is commonly used when one is looking for patterns in interviews and comparing different experiences. An interview guide was developed a priori to reflect the purposes of this study. The first author then completed 19 interviews via telephone with 2 more performed in-person using the interview guide. All interviews were digitally recorded, transcribed verbatim, checked for accuracy, and uploaded into a qualitative data software program, NVivo 11 Pro Software (QSR International, 2017). NVivo allows you to store, label, and categorize large quantities of narrative data to describe common experiences that the participants are elucidating.

Analysis involved reading all transcripts as a unit first, rereading, and then labeling segments of text that reflected similar ideas or experiences. A coding frame (method of categorizing the content) was developed so that the similar ideas or experiences could be successively labeled, defined, and sorted. Constant comparison technique was used as transcripts were compared with one another, and codes were added if new ideas emerged from successive transcripts. The initial transcripts were reviewed again and relabeled or recoded in this iterative process until no new ideas or experiences were found. In second-level analysis, narrative data were analyzed per coded groups to form categories of information that combined to form themes that aligned with the research purpose. Finally, recoding was performed 6 months after the original coding to check for intersubjectivity (or how close the results were to one another), and results were found to be consistent, supporting validity and reliability of the findings.

## Results

### Participant Characteristics

A total of 151 potential subjects were identified, of which 28 were enrolled. Of those who were not enrolled, 76 did not return messages, 24 were ineligible, 17 could not be reached, and 6 were lost to follow-up. There were 7 participants randomized to the mHealth group, 11 in the home equipment group, and 10 in the SoC group. We had attrition of 3 subjects before completing their 12-week enrollment, 1 from the mHealth group and 2 from the in-home equipment group citing they were too busy or sick to continue, leaving 25 participants at study conclusion. We aimed to enroll 60 participants to be able to compare each group. Due to limitations in the funding period and 1 clinic reorganizing management, we did not meet this goal and thus are not powered sufficiently to determine between-group differences via inferential statistics; however, within-group changes from pre to post survey were tested to indicate feasibility for future clinical trial(s).

All participants had a diagnosis of chronic HF, and this was confirmed by medical record audit. Of note, our participants’ educational attainment is below national averages (eg, 18% [5/28] with bachelor’s degree), with substantial comorbid conditions such as 32% (9/28) with diabetes and 68% (19/28) with hypertension. Table 3 reflects the general demographics of the participants.

### Results of Survey Data

We asked about home monitoring of BP, weight, and symptoms. At baseline, 29% (8/28) of participants did not monitor their BP, 46% (13/28) did not monitor their weight, and 68% (19/28) did not monitor for other symptoms at home. Postintervention data demonstrated that 100% of both the mHealth and in-home groups were monitoring their BP and weight, whereas, only 75% (6/8) of the SoC group monitored their BP and 88% (7/8) monitored their weight—although not often on a daily basis. We report frequencies and trends in differences between groups; see Table 4.

**Table 3:** Study demographics (baseline N=28).

Characteristic		n (%)
**Age (years)**		
	55-59	5 (18)
	60-64	6 (21)
	65-69	7 (25)
	Above 70	10 (36)
**Gender**		
	Female	12 (43)
	Male	16 (57)
**Race/ethnicity**		
	Black, non-Hispanic	5 (18)
	White, non-Hispanic	22 (79)
	American-Indian/Alaskan native	1 (4)
**Educational level**		
	Some high school/high school graduate/GED (General Educational Development)	11 (39)
	Some college/associate’s degree	12 (43)
	Bachelor’s degree	5 (18)
**Marital status**		
	Single/never married	3 (11)
	Married	13 (46)
	Separated/divorced/widowed	12 (43)
**Chronic conditions**		
	Diabetes	9 (32)
	Asthma	3 (11)
	Lung disease	12 (43)
	Heart disease	28 (100)
	Hypertension	19 (68)
	Arthritis or other rheumatic disease	15 (54)
	Cancer	8 (29)

**Table 4:** Home monitoring.

Characteristic			Baseline			Post intervention		
			mH^a^ (n=7)	HE^b^ (n=11)	SoC^c^ (n=10)	mH (n=6)	HE (n=9)	SoC (n=10)
**Do you currently monitor your blood pressure, weight, or other health symptoms related to your risk for heart failure at home?, n (%)**								
	Yes		5 (71)	10 (91)	7 (70)	6 (100)	9 (100)	8 (80)
	No		2 (29)	1 (9)	30 (3)	0 (0)	0 (0)	2 (20)
**Which of the following do you currently monitor at home?, n (%)**								
	**Blood pressure**							
		Yes	5 (71)	10 (91)	5 (50)	6 (100)	9 (100)	6 (75)
		No	2 (29)	1 (9)	50 (5)	0 (0)	0 (0)	2 (25)
	**Weight**							
		Yes	2 (29)	7 (64)	6 (60)	6 (100)	9 (100)	7 (88)
		No	5 (71)	4 (36)	4 (40)	0 (0)	0 (0)	1 (13)
	**Health symptoms**							
		Yes	3 (43)	5 (45)	1 (10)	3 (50)	8 (89)	7 (88)
		No	4 (57)	6 (55)	9 (90)	3 (50)	1 (11)	1 (13)

^a^mH: mobile health group.

^b^HE: home equipment group.

^c^SoC: standard care group.

#### General Health and Health Distress

On the health and health distress scales, at baseline, 50% (14/28) of the participants rated their health as poor or fair, 25% (7/28) and 36% (10/28) were very often/always discouraged and frustrated by their health, with 21% (6/28) very often/always fearful for their health. Only the mHealth group trended toward a change in very often/always discouraged by their health, with 43% (3/7) reporting discouragement at baseline and 0% post intervention (0/6). In our exploratory analysis for our feasibility aim, we examined if our interventions with the older participants living with HF added to their distress. A repeated measures analysis of variance showed a marginally significant main effect of time for the health distress items (*F*_1,22_=4.080; *P*=.056; *η*^2^=0.156; power=0.489), indicating that overall, participants’ scores showed less health distress (ie, discouragement, fear, worry, and frustration) in their last assessment (mean 2.01, SD 0.81) than in their first assessment (mean 2.36, SD 1.24). Moreover, 15.6% of the variability among our observations can be attributed to time. See Table 5.

#### Self-Care and Provider Care Perceptions

The Self-Care Perceptions scale indicated that 89% (25/28) of participants were confident about their ability to care for their health at baseline, with 79% (22/28) strongly/somewhat agreeing that they should decide how to manage and control their health (see Table 6). However, when we queried about provider care perceptions, the majority of the participants believed that the provider/doctors should be managing their health rather than themselves, and 71% (21/28) reported that one should trust and not question the doctor or nurse. However, when we look at group differences, the mHealth equipment users seemed to become more empowered as they relied more on themselves post intervention. These changes were not mirrored in the home equipment or SoC group (see Table 7).

**Table 5:** Self-rated health and health distress scales.

Characteristic		Baseline			Post intervention		
		mH^a^ (n=7)	HE^b^ (n=11)	SoC^c^ (n=10)	mH (n=6)	HE (n=9)	SoC (n=10)
**In general, would you say your health is, n (%)**							
	Excellent/very good	2 (29)	2 (18)	2 (20)	1 (17)	1 (11)	2 (20)
	Good	3 (43)	2 (18)	3 (30)	3 (50)	3 (33)	5 (50)
	Fair/poor	2 (29)	7 (64)	5 (50)	2 (33)	5 (56)	3 (30)
**How much time during the past 2 weeks were you discouraged by your health problems?, n (%)**							
	Very often/always	3 (43)	3 (27)	1 (10)	0 (0)	1 (11)	1 (10)
	Sometimes	2 (29)	3 (27)	3 (30)	2 (33)	4 (44)	5 (50)
	Never/seldom	2 (29)	5 (45)	6 (60)	4 (67)	4 (44)	4 (40)
**How much time during the past 2 weeks were you fearful about your health?, n (%)**							
	Very often/always	2 (29)	3 (27)	1 (10)	0 (0)	2 (22)	2 (20)
	Sometimes	0 (0)	0 (0)	1 (10)	0 (0)	1 (11)	2 (20)
	Never/seldom	5 (71)	8 (73)	8 (80)	6(100)	6 (67)	6 (60)
**How much time during the past 2 weeks was your health a worry to your life?, n (%)**							
	Very often/always	1 (14)	2 (18)	1 (10 )	0 (0)	0 (0)	1 (10)
	Sometimes	0 (0)	1 (9)	2 (20)	1 (17)	3 (33)	1 (10)
	Never/seldom	6 (86)	8 (73)	7 (70)	5 (83)	6 (67)	8 (80)
**How much time during the past 2 weeks were you frustrated by your health problems?, n (%)**							
	Very often/always	3 (43)	5 (45)	2 (20)	0 (0)	1 (11)	1 (10)
	Sometimes	1 (14)	1 (9)	2 (20)	3 (50)	5 (56)	2 (20)
	Never/seldom	3 (43)	5 (45)	6 (60)	3 (50)	3 (33)	7 (70)

^a^mH: mobile health group.

^b^HE: home equipment group.

^c^SoC: standard care group.

**Table 6:** Self-care perceptions.

Characteristic		Baseline			Post intervention		
		mH^a^ (n=7)	HE^b^ (n=11)	SoC^c^ (n=10)	mH (n=6)	HE (n=9)	SoC (n=10)
**The way I care for my health is important to me, n (%)**							
	Strongly/somewhat agree	6 (86)	10 (91)	10 (100)	5 (83)	8 (89)	9 (90)
	Neutral	0 (0)	1 (9)	0 (0)	1 (17)	1 (11)	0 (0)
	Somewhat/strongly disagree	1 (14)	0 (0)	0 (0)	0 (0)	0 (0)	1 (10)
**I am confident about my ability to care for my health, n (%)**							
	Strongly/somewhat agree	6 (86)	11 (100)	8 (80)	5 (83)	9 (100)	10 (100)
	Neutral	0 (0)	0 (0)	1 (10)	1 (17)	0 (0)	0 (0)
	Somewhat/strongly disagree	1 (14)	0 (0)	1 (10)	0 (0)	0 (0)	0 (0)
**I can decide on my own how to go about managing my health, n (%)**							
	Strongly/somewhat agree	6 (86)	9 (82)	7 (70)	5 (83)	5 (56)	7 (77)
	Neutral	1 (14)	1 (9)	0 (0)	0 (0)	0 (0)	0 (0)
	Somewhat/strongly disagree	0 (0)	1 (9)	3 (30)	1 (17)	4 (44)	3 (30)
**I have a great deal of control over what happens to my health, n (%)**							
	Strongly/somewhat agree	5 (71)	8 (73)	9 (90)	5 (83)	7 (78)	9 (90)
	Neutral	1 (14)	0 (0)	1 (10)	0 (0)	1 (11)	0 (0)
	Somewhat/strongly disagree	1 (14)	3 (27)	0 (0)	1 (17)	1 (11)	1 (10)

^a^mH: mobile health group.

^b^HE: home equipment group.

^c^SoC: standard care group.

**Table 7:** Provider care perceptions.

Characteristic		Baseline			Post intervention		
		mH^a^ (n=7)	HE^b^ (n=11)	SoC^c^ (n=10)	mH (n=6)	HE (n=9)	SoC (n=10)
**Except for serious illness, it is better to take care of your own health than to seek professional help, n (%)**							
	Strongly/somewhat agree	2 (29)	5 (45)	4 (40)	2 (33)	3 (33)	3 (30)
	Neutral	0 (0)	0 (0)	1 (10)	0 (0)	0 (0)	1 (10)
	Somewhat/strongly disagree	5 (71)	6 (55)	5 (50)	4 (67)	6 (67)	6 (60)
**It is better to rely on the judgments of doctors (who are experts) than to rely on “common sense” in taking care of you own body, n (%)**							
	Strongly/somewhat agree	6 (86)	5 (45)	6 (60)	3 (50)	7 (78)	9 (90)
	Neutral	0 (0)	1 (9)	1 (10)	0 (0)	2 (22)	0 (0)
	Somewhat/strongly disagree	1 (14)	5 (46)	3 (30)	3 (50)	0 (0)	1 (10)
**Recovery is usually quicker under the care of a doctor or nurse than when patients take care of themselves, n (%)**							
	Strongly/somewhat agree	5 (71)	11 (100)	10 (100)	4 (67)	9 (100)	10 (100)
	Neutral	1 (14)	0 (0)	0 (0)	2 (33)	0 (0)	0 (0)
	Somewhat/strongly disagree	1 (14)	0 (0)	0 (0)	0 (0)	0 (0)	0 (0)
**It is better to trust the doctor or nurse in charge of a medical procedure than to question what they are doing, n (%)**							
	Strongly/somewhat agree	5 (71)	8 (73)	7 (70)	3 (50)	4 (44)	10 (100)
	Neutral	1 (14)	0 (0)	1 (10)	1 (17)	1 (11)	0 (0)
	Somewhat/strongly disagree	1 (14)	3 (27)	2 (20)	2 (33)	4 (44)	0 (0)
**I’d rather be given many choices about what’s best for my health than have the doctor make the decisions for me, n (%)**							
	Strongly/somewhat agree	5 (71)	10 (91)	10 (100)	4 (67)	5 (56)	8 (80)
	Neutral	0 (0)	0 (0)	0 (0)	2 (33)	0 (0)	0 (0)
	Somewhat/strongly disagree	2 (29)	1 (9)	0 (0)	0 (0)	4 (44)	2 (20)

^a^mH: mobile health group.

^b^HE: Home equipment group.

^c^SoC: standard care group.

**Table 8:** Patient communication and engagement.

Characteristic		Baseline			Post intervention		
		mH^a^ (n=7)	HE^b^ (n=11)	SoC^c^ (n=10)	mH (n=6)	HE (n=9)	SoC (n=10)
**How often does your health care team listen carefully to you?, n (%)**							
	Very often/always	4 (57)	9 (82)	10 (100)	3 (50)	8 (89)	9 (90)
	Sometimes	2 (29)	1 (9)	0 (0)	3 (50)	0 (0)	1 (10)
	Never/seldom	1 (14)	1 (9)	0 (0)	0 (0)	1 (11)	0 (0)
**How often does your health care team explain in a way you can understand?, n (%)**							
	Very often/always	5 (71)	11 (100)	9 (90)	4 (67)	8 (89)	10 (100)
	Sometimes	1 (14)	0 (0)	1 (10)	1 (17)	1 (11)	0 (0)
	Never/seldom	1 (14)	0 (0)	0 (0)	1 (17)	0 (0)	0 (0)
**How often does your health care team show respect for what you say?, n (%)**							
	Very often/always	4 (57)	8 (73)	10 (100)	4 (67)	8 (89)	10 (100)
	Sometimes	2 (29)	2 (18)	0 (0)	2 (33)	1 (11)	0 (0)
	Never/seldom	1 (14)	1 (9)	0 (0)	0 (0)	0 (0)	0 (0)
**How often does your health care team spend enough time with you?, n (%)**							
	Very often/always	5 (71)	10 (91)	10 (100)	6 (100)	8 (89)	9 (90)
	Sometimes	1 (14)	1 (9)	0 (0)	0 (0)	1 (11)	1 (10)
	Never/seldom	1 (14)	0 (0)	0 (0)	0 (0)	0 (0)	0 (0)

^a^mH: mobile health group.

^b^HE: home equipment group.

^c^SoC: standard care group.

#### Patient Communication and Engagement

We asked about the participant’s communication and engagement with providers. Overall, participants indicated that they experienced good communication/engagement with providers, which did not change appreciably after 12 weeks with satisfaction scores ranging from 80% to 92% from pre intervention to post intervention. See Table 8.

#### Monitoring Adherence

We asked participants about adherence to self-monitoring for signs and symptoms of HF complications at baseline and post intervention. All but 3 of the participants told us that they had received instructions from their provider about monitoring for signs and symptoms of HF complications (these 3 were excluded from further questions), Questions were concerned with how often did you forget or were careless or stopped monitoring your signs and symptoms. At baseline, 44% (11/25) sometimes or very often forgot to monitor, 40% (10/25) were careless, and 52% (13/25) stopped monitoring when they felt better. Postsurvey data revealed that 100% (15/15) of participants receiving either the mHealth or home equipment reported never/seldom forgetting to monitor their symptoms daily, a 50% increase from baseline. In the standard care group, 67% (6/9) of participants reported never/seldom forgetting to monitor their daily symptoms at baseline, whereas post intervention, this number decreased to 22% (2/9). At baseline, 50% (8/16) of mHealth and home equipment users reported they sometimes/very often/always stopped monitoring their symptoms when feeling better. This number decreased to 14% (2/15) post intervention. The SoC group remained unchanged from baseline to post intervention, with 55% (5/9) of participants reporting they sometimes/very often/always stopped monitoring when feeling better (data not included in tables). Furthermore, a Pearson chi-square test on data captured post intervention showed a trend toward significance (*χ* ²_4)_ =7.852; *P*=.097), indicating that group membership and monitoring adherence were associated.

#### Technology and Equipment Usability Survey

For the technology anxiety portion of the pre and post survey, 36% (10/28) and 32% (9/28) of our older adults indicated that technology made them nervous or fearful at baseline without significant change post intervention. For equipment usability and self-management, 12 of 15 participants ranked equipment usability at 90 points or above (on a 100-point scale), with an overall mean score of 91.1. The mean score was 84.2 for mHealth users and 95.8 for the home equipment group. When asked about the feasibility of using the mHealth and home equipment regularly to monitor their symptoms, 93% (14/15) of participants agreed that the equipment was easy enough to use on a daily basis.

**Table 9:** Summary findings using mean baseline and postintervention scores.

Scale	Baseline			Post intervention		
	mH^a^ (n=7), mean (SD)	HE^b^ (n=11), mean (SD)	SoC^c^ (n=10), mean (SD)	mH (n=6), mean (SD)	HE (n=9), mean (SD)	SoC (n=10), mean (SD)
Health distress	2.40 (1.04)	2.39 (1.38)	2.14 (1.06)	1.75 (0.47)	2.11 (0.85)	2.08 (0.96)
Self-care perceptions	4.05 (0.91)	4.45 (0.69)	4.19 (0.48)	4.46 (0.98)	4.22 (0.51)	4.20 (0.61)
Provider care perceptions	2.60 (0.87)	2.69 (0.55)	2.60 (0.57)	2.77 (0.95)	2.22 (0.78)	2.22 (0.65)
Communication/engagement	4.30 (0.74)	4.36 (0.57)	4.61 (0.31)	4.21 (0.87)	4.64 (0.75)	4.60 (0.47)
Monitoring adherence	3.80 (1.50)	3.55 (0.82)	3.78 (0.85)	4.72 (0.53)	4.37 (0.61)	3.56 (1.07)
Technology anxiety	2.00 (0.79)	3.18 (1.23)	2.00 (1.32)	1.75 (0.88)	2.83 (1.32)	1.75 (1.14)

^a^mH: mobile health group.

^b^HE: home equipment group.

^c^SoC: standard care group.

**Table 10:** Symptoms of heart failure scale (SSQ-HF).

During the past week did you have	Week 1 percentage positive^a^ (N=17)	Week 6 percentage positive (N=15)	Week 12 percentage positive (N=14)
Shortness of breath during the daytime	35	33	57
Shortness of breath when you lay down	6	7	21
Fatigue or lack of energy	53	53	57
Chest pain	0	0	14
Leg or ankle swelling	35	27	36
Difficulty sleeping at night	41	20	14
Dizziness or loss of balance	35	40	39

^a^Percentage positive=percentage of participants reporting symptoms that week.

### Summary of Survey Findings Using Mean Scores

The mean scores of all surveys at baseline compared with post intervention in each group have been presented in Table 9. As noted earlier, sample sizes were not sufficient to show statistical significance, although trends in differences before and after the intervention may be clinically meaningful and provide estimates for future study.

### Weekly Symptom and Health Status Survey

We made 289 telephone calls to the participants over 12 weeks. For the equipment groups (mHealth and home equipment), we checked for common HF symptoms, provider/hospital visits, and lifestyle and activity patterns. For the standard care patients, we only queried about provider/hospital visits.

For HF symptoms, multiple symptoms were experienced each week. They varied throughout the study with no discernible patterns emerging. Table 10 (Symptoms of heart failure scale; SSQ-HF [29]) shows the percentage of subjects reporting symptoms cross-sectionally at 3 time points: week 1-baseline, week 6-midpoint, and week 12-completion. We asked 4 questions weekly about improvements and changes in symptoms, BP, and weight. Overall symptom improvements were reported 48 times (out of 177 or 27% of the time), with participants describing less fatigue and higher energy levels or improved sleep, less dizziness, reduced swelling, and medication changes, again demonstrating waxing and waning of symptoms from week to week. In the home equipment group, we asked about changes in BP and weight. Overall, 20% of the home equipment participants reported changes in BP or weight during the 12 weeks. Finally, we asked about provider and ED visits; there were 79 provider visits in 12 weeks with 7 visits to the ED (mean of 3.4 visits per person). There were no differences found between groups.

### Equipment Status and Issues

We asked each week about equipment problems, and 23.2% (41/177) of the time, problems were reported. Most common complaints were about the automated wrist BP cuff in the home equipment group, the scale reading slightly different weights when repeated measures were taken by the participants, and mHealth equipment connectivity problems (the Bluetooth function of the scales). We asked if participants’ needed us to retrain them in using their equipment and did this 4.5% (8/177) of the time, home equipment users answered affirmatively 2 times, and there were 6 requests from the mHealth users. Interestingly, there were essentially an equal number of problems with home equipment when compared with mHealth internet connected equipment.

### Lifestyle Behaviors

We asked a few questions each week about lifestyle adherence behaviors of diet, medication, and exercise. Overall, 67% (19/28) of the participants indicated they were on a low-sodium diet, although the methods of this diet varied greatly and most reported they just “did not add salt” to most of their food (but did not necessarily buy low-sodium foods). For adherence to medications, 97% (27/28) told us they were adherent; however, 15% (4/25) later said they were skipping medications some days, with no differences seen between groups. When asked about participation in exercise, 68% (19/28) of the participants reported they were exercising each week; however, when asked what type of exercise, many participants included walking “every now and then,” “getting out of the house,” house cleaning, or physical therapy as qualifiers for exercise. Other exercise regimens included weekly exercise classes at a fitness center, stationary biking, strength training, and taking daily 30-min walks. There were no differences seen between groups.

### Nurse Call Center Data and Adherence to Daily Readings

The call center nurses received 872 total mHealth readings throughout the 12-week study, 500 for BP and 372 for weight, demonstrating that participants were largely adherent to our instructions with an 85.15% (872/1024) overall adherence rate. Of those readings, 50 triggered alerts, meaning the submitted readings fell outside doctor-provided parameters. BP readings accounted for 30 alerts, 26 of which resulted in 14 triage calls. However, most of these were to 1 participant who had multiple alerts for high BP readings. Of the 4 alerts remaining that were not triaged, 2 were due to mHealth equipment error and 2 due to call center nurse error. Moreover, 2 abnormal BP readings failed to trigger any alerts; reasons for this remain undetermined, but this may have been probably due to a connectivity issue. Weight gain triggered 20 alerts and 6 triage calls. Of the 14 alerts not triaged, 11 were related to equipment problems (the call center was aware of this) and 3 were errors by the call center. A total of 7 weight readings did not trigger alerts for reasons undetermined.

During the 12 weeks, 224 readings were missing, 71 for BP and 153 for weight. Call center nurses phoned participants when daily readings were not transmitted. Moreover, 2 participants were responsible for 168 of the missed readings, 1 who suffered a leg injury and could not weight-bear to stand on the scales and 1 participant who was essentially nonadherent due to continued mHealth equipment tampering by grandchildren (ie, disassembling). Call center nurses were instructed to discontinue calls to these participants. Of the remaining 56 missed readings, call center nurses placed 50 reminder calls to participants, and there were 6 instances where calls were not placed due to error. Ultimately, 89% of the time, patients were contacted correctly for missed readings.

### Home Equipment Group Daily Log and Monitoring Adherence

The home equipment group was instructed to take daily BP and weight measures and record them in a log provided by the study team. Out of the 11 participants in this group, 4 (36%, 4/11) did not return their logs as per protocol; we received 1137 readings (62% adherence rate). The log data demonstrated the results were out of range, similar to the mHealth group, 63 times for BP and 60 times for weight; that is, alerts would have been triggered for these same values in the mHealth group participants. Although we did not collect information on whether the providers were contacted by the participants as a result of these physiologic changes, participants may have needed intervention from their provider.

### Qualitative Results

Of the 21 participants engaged in the qualitative study, 9 were in the home equipment group, 6 in the mHealth group, and 6 in standard care group. We identified 4 key themes from the narrative analysis, *Communication and Engagement with Health Care Providers*, *Home Monitoring with Technology*, *Awareness of the Importance of Self-Monitoring and Management*, and *Persistent Health Problems*. Themes were based on the analysis of the narratives, which demonstrated repetition of common participants’ experiences. The themes, categories, and exemplary narratives that support each are included in Table 11.

#### Theme 1: Traditional Communication and Engagement With Health Care Providers

Most participants were satisfied with their established methods of communication with their providers, specifically, they used phone calls to the clinic and in-person clinic visits during office hours, although several had the burden of arranging for transportation. Communication and engagement were specified in 3 ways: health care system issues, both good and bad provider communication, and by routine ED visits. Only 1 participant utilized the communication internet portal that was provided by the health care system. All other participants did not envision other methods of care communication other than traditional phone calls or in-person visits. As seen in Table 11, narratives described health care system problems and both good and poor communication with providers. Unexpectedly, participants described using the ED as a routine practice for accessing care. Narratives describing self-management of their HF symptoms were uncommon before their participation in this study, participants would wait until a symptom was unmanageable and then relied on their provider to take care of the problem.

#### Theme 2: Home Monitoring With Technology

When questioned about home monitoring with the mHealth equipment or the home equipment, they described it by 3 categories: as helpful, problematic, and for the mHealth equipment participants, “like someone was watching over me.”

**Table 11:** Results from qualitative analysis: themes, categories, and participant narratives. Numbers that follow the narrative represent distinct study participants and group to which they were randomized: mHealth (mobile health) connected technology, home equipment, or standard of care.

Themes and categories		Narrative
**Traditional communication and engagement with health care providers**		
	Health care system problems	“I actually made a formal complaint to the hospital. I don’t know how many calls I’ve made and they essentially said ‘well there is nothing we can do about it.’ and I said, ‘Well there is something I can do about it, I can go somewhere else...’” [Participant 14, mHealth]
	Provider communication: good	“They usually call us back in the next 30 minutes or an hour. You don’t get nobody, I mean, when you call, you just have to leave a message. But usually, they call you back in the next 30 minutes to an hour. But they are good to us, they are very good to us... [The communication] is pretty good.” [Participant 16, home equipment]
	Provider communication: poor	“If I’m feeling that bad now, then I want to see a doctor. I need to see a doctor now. Not 2 or 3 weeks from now when I might be feeling fine. I’m feeling so bad now, I want to see what’s going on. I want you to see me now” [Participant 27, home equipment] “[is it easy to communicate with your doctor] No, it’s really not...I didn’t want it to get really bad, so I had an appointment with my heart doctor...they wanted me to make an appointment and come back in...and I didn’t want to...because it takes several hours to do that.” [Participant 22, home equipment]
	Emergency room visits are routine	“I went to Emergency. Yeah. They are really, really good here and it’s much quicker than anything else.” [Participant 1, home equipment] “I went twice this month [to the ER], I didn’t go last month.” [Participant 23, standard care]
**Home monitoring with technology**		
	Helpful	“It helped me with my blood pressure and my weight, It told me what I needed, you know.” [Participant 18, mHealth] “I think that it’s a good thing and that it would help people that live a distance away because they see that there is a problem, that they can either contact the doctor’s office or get up to the hospital as quick as they can. It doesn’t make me nervous or anything, I am used to this stuff [technology].” [Participant 11, mHealth]
	Problematic	“I didn’t like the equipment. It was ok except for the scales. It was so hard to set it up and everything to get the weight. By the time you turned the iPad on, got the scale on the floor on a level spot, pushed the button underneath it to get it to weigh you, the iPad had kicked off, and by the time you reset it, the scales kicked off. So you literally had to have someone help you do it.” [Participant 12, mHealth]
	Watching over me	“Well that was good, knowing that somebody was there, watching over it, who actually knew something about medicine. It was kind of a plus.” [Participant 14, mHealth] “It was good. It didn’t bother me none. I liked people checking, you know, to see how I was doing.” [Participant 18, mHealth]
**Patient awareness of the importance of self-monitoring and management**		
	Symptom surveillance	“...it keeps my mind focused on what I have to eat and if I eat this stuff with too much salt...it is going to make me have to retain fluid...you’re stuck with a situation where you can’t take a breath of air, you know, I couldn’t even blow my nose. My lungs were being squished so much that I couldn’t even take a breath enough to blow my nose.” [Participant 9, home equipment] “The equipment helps...you know if you gained weight overnight you know to take Lasix. If I’m about 4 or 5 pounds over, I take a little more Lasix.” [Participant 17, home equipment]
	Becoming a routine practice	“It brought a level of comfort ...a baseline reading, kind of what was normal for me. Then if I saw something abnormal, I would try to identify what did I do?...So it gave me an idea of what was causing the changes. But, yeah, it did help. It made me more aware of my own health... I got in the habit of taking my blood pressure every day.” [Participant 3, home equipment] “…because it gets you used to monitoring yourself and then you start realizing just what it means when you see them numbers off...never did realize before how much difference it made.” [Participant 25, home equipment]
**Persistent health problems**		
	Uncertainty	“...changed my blood pressure medicine after the congestive heart failure episode. They increased my blood pressure, changed it, and increased it, he thought after that, that it was probably the diuretic that was causing the problem. Or maybe, I don’t know, may have been the heart, the blood pressure medicine. The Lasix should have gotten rid of it, so, anyway it didn’t go away...So I’m not sure what’s going on.” [Participant 1, home equipment]
	Frustration	“I just felt like it was just too much with...And nurses were coming in to check me out make sure that everything was going right and just seemed like a lot was going on and I thought I’d just go ahead and drop out of this [study].” [Participant 2, mHealth]

Exemplary narratives that described their perspectives about the monitoring technology are listed in Table 11. Findings from narratives supported that most participants in the equipment groups appreciated the equipment and found it helpful in monitoring for signs and symptoms of decompensation. An exemplary narrative:

I think you would have probably found this weight gain, Yeah, if you were monitoring the scale, you would have seen. I think it’s probably happened over a 10, 15 day period. So, I think it would have been very evident had I been one of the ones that got the telemetry

Participant 1; home equipment

Several participants discussed that monitoring increased their confidence in themselves, and they were less fearful of HF exacerbations. However, 2 participants in the equipment groups did not like or want to monitor their BP or weight at home, instead they recognized impending crises by other methods, such as feeling “dizzy, tired, or short-of-breath,” and as a result, they self-treated with a diuretic or visited their provider or ED for care. For example:

If I get real short of breath, I mean, I call the doctor. Generally, if my fluid is building up, I have to take an extra Lasix.”

Participant 25, home equipment

When these and the standard care participants were asked why they did not home-monitor before this study, most replied that they did not own a scale or BP cuff, they were not interested, or they did not see the need. For instance:

I’ve weighed the same for about 12 years, so I don’t need to check it

Participant 8, standard care

I don’t have the equipment for one thing and if I did, I really don’t know how to run it.

[Participant 26, standard care].

Narratives demonstrated that the mHealth group experienced a feeling of security and alliance to our research team that was very encouraging to them. For example:

it brought a level of comfort to me...it was like a friend calling rather than an annoyance...to know if I am in trouble or if I have a new problem...it is a wonderful idea

Participant 2, mHealth

#### Theme 3: Awareness of the Importance of Self-Monitoring and Management

Participants in the 2 equipment groups overwhelmingly reported that they became more aware of the importance of monitoring their weight, BP, and symptoms because of this study. Narratives described that symptom surveillance became a routine practice during the 12 weeks of this study that was likely to prevail going forward. Reasons expressed that our weekly reiteration of the symptom survey and status check taught them which symptoms to watch for and encouraged them to self-manage. For example:

I never thought of whether my feet get swollen or not...then they asked me almost every week when they called, now I know to watch for it

Participant 6, mHealth

An exemplary narrative:

The whole thing just makes you realize that you’ve got to keep a close eye on what your weight and blood pressures because those are the factors that are going to get you...it is so important that I do it every day.

Participant 9, home equipment

#### Theme 4: Persistent Health Problems

Participants repeatedly told us they suffered from persistent health problems and challenges associated with their HF, although this was not included as part of the interview guide questions. They described continuous uncertainty and frustration from living with chronic HF and described instances that were traumatizing to them:

People don’t understand how it feels, that I couldn’t even bend over to tie my shoes. It was the hardest thing for me to do...everything in there is so full, your belly is full and you haven’t even eaten, I would have to stop trying and take a breath.

[Participant 9, home equipment].

See Table 11 for other narratives.

## Discussion

### Principal Findings

The main aim of this mixed-methods study was to examine the feasibility of older adults with HF using connected mHealth technologies at home. Specifically, we looked at patients’ ease and satisfaction with the equipment, communication patterns with their providers, and their engagement in HF self-monitoring and management as an essential first step before designing a clinical trial to test such an intervention. Results demonstrated the feasibility of older adults with HF using the equipment, completing the surveys, maintaining study engagement, and even improving self-management. Interviews augmented the survey data by providing synergistic information that helped clarify and explain the survey data and should be considered a strength of this study.

Before this study, participants more often monitored their BP rather than their weight at home, and most did not monitor for symptoms. We also discovered that this monitoring was not done routinely or on a daily basis for most of the participants despite their poor health status and persistent symptoms. They often forgot or skipped days, especially when they felt better. Postintervention data on monitoring adherence showed a change in consistent monitoring to 100% in both of the equipment groups for daily self-monitoring, and 100% said they did not forget to monitor or skip days. In the SoC group, 78% of the time, they forgot to monitor their symptoms post intervention. This is an important finding partially explained by our interview data, which may demonstrate that the equipment groups, through training and recurring messages given by our weekly status calls, increased knowledge and skill development in self-management. It is established that HF, like many other chronic diseases, can be better managed when the patient exhibits self-care behavior [9]. HF self-management involves lifestyle adjustments [10] and physiological monitoring and surveillance [9]. It is frequently reported that these are difficult skills for HF patients to acquire because of the complexity of their treatment plan, cognitive changes, poor health literacy, and patient inclinations [9]. Individuals with HF most often have multiple comorbid conditions, affecting their ability to manage this multiplicity; thus, the interventions for the comorbidities must be a priority as well [31].

This study showed that the general health of our participants was mostly fair or poor at baseline with no real differences between the groups, which is consistent with data demonstrating poor quality of life in these individuals [9]. They reported discouragement, frustration, and fear regarding their health, emotions that reduce quality of life. However, post intervention, the mHealth group showed less discouragement, fear, and frustration regarding their health. This finding is supported by other research describing that home telehealth is found to increase quality of life for these individuals [32].

We aimed to examine perspectives of these older adults while they used the equipment and self-managed their HF. Although older adults may require training on technology literacy [33], mHealth monitoring is designed to foster autonomy and independence in chronic disease self-management and may have improved their feelings of distress. Research demonstrates similar responses in a study of community-dwelling older adults with poor health as they similarly shared feelings of powerlessness that emerged during the process of regaining independence [34]. Not surprising in these older adults, about one-third of them were nervous or fearful about using technology; however, when asking our participants about whether they thought home monitoring was feasible, easy to use, and helped them manage their symptoms, equipment usability and ease both scored in the 90 percentiles (out of 100), indicating they would use this on a daily basis. A 2017 report from the Pew Foundation found that the percentage of older adults using technology, such as the internet, smartphones, tablets, and social media, has increased steadily since 2000 [35]. Seniors who are more affluent and have a higher educational level have similar rates of use to adults younger than 65 years [35]. Our baseline findings related to fear of using the technology (36%) may be, in part, due to our participants’ educational levels (18% bachelor’s degree). Even though use of technology is increasing, the Pew Report stated that 73% of older adults say “they need someone else to set it up or show me how to use it” [35]. These findings are consistent with other studies related to utilization rates in older adults to monitor HF [36] and positive perceptions of use of technology to monitor HF [37].

We found that our participants ranked communication with their providers as quite satisfactory using traditional methods of phone calls and clinic visits, although qualitative data described participants having both good communication and substandard experiences with communication and obtaining access to care. mHealth technology is supported as a method to increase communication and access to care [38,39]. It was interesting that we found the participants mostly relied on their provider to manage their health—instead of themselves. Under the traditional model of health care, older adults are more accustomed to a paternalistic approach and interact with providers in a clinic or hospital setting to meet their needs. The dependency initially indicated from results on the provider care perception survey and decreasing post intervention may actually reflect a transition from the traditional model of health care to a more decentralized environment [40,41]. Research supports that HF patients will experience improved quality of life [42] and positive behavioral changes in BP and weight monitoring as they demonstrate confidence for self-management of HF [41,42].

The weekly symptoms and health status survey provided insights into symptoms, adherence to lifestyle recommendations, and provider visits. Our participants experienced persistent struggles with distressing HF symptoms that exhibited no pattern in this study. Interview data described uncertainty and frustration with ongoing symptoms. However, the participants described that home monitoring was helpful and those that had mHealth equipment described a feeling of security knowing someone was available to them. This phenomenon has been reported by others [43].

We also wanted some insight into how these patients were currently managing their HF at home. National HF guidelines recommend developing a care management plan to include appropriate levels of physical activity and dietary adherence, especially for sodium intake [44,45]. Studies demonstrate that lower sodium intake is associated with improved status in those with symptomatic HF [46]; however, HF patients have difficulty adhering to this restriction [47]. Moreover, 67% of our participants self-reported a low-sodium diet, which is quite high given the objective measure of approximately 34% in a recent study [46]. In this study, we asked about these lifestyle parameters weekly in both equipment groups and found that self-reported physical activity was also likely overstated, similar to other studies that measure with self-reported questionnaires [48]. For example, Yates et al report that 38% of HF patients self-reported meeting physical activity recommendations, but when measured objectively, 0% met recommendations.

Gilorta et al [49] found HF knowledge gaps that were identified through surveys given to HF patients post discharge, along with identifying reasons for nonadherence. Patients reported that (1) they did not know they had HF despite being informed of this diagnosis while in the hospital or (2) they strayed from their strict dietary restrictions as they felt better and did not have symptoms. Similar patterns were reported in this study. The Heart Failure Society of America Guidelines recommend continuing education over time because of lack of efficacy with a single educational session that commonly occurs during a hospital discharge [49]. Telehealth interventions have potential for real-time education and symptom support in addition to monitoring for physiological alterations [50].

The nurse call center received 872 readings, and we completed 289 telephone calls following our participants on a weekly basis. Overall, we found that the call center triaged the calls accurately with only a few errors made. We had patients in very rural areas but had only a few missed transmissions, likely a result of cellular connection interruption. From these data, it appears that it is quite feasible to use this technology in rural as well as urban areas.

Our participants reported 79 provider visits and 7 ED visits in 12 weeks, that is, an average of 3.44 visits per person. HF is a syndrome that places a substantial burden on the health care system as well as the patient [1], with over 1 million hospitalizations annually and estimated 7.4 million ED visits [51]. The Centers for Medicare and Medicaid require public reporting of HF admissions and have established penalties for hospitals with high readmission rates. Primary reasons for ED visits are breathing difficulties (88% of patients) followed by chest discomfort (35%) and fatigue (16%) [52]. Recently, mHealth was supported for reducing hospital readmissions [32,53,54] but not necessarily ED visits [51].

### Study Limitations

With this study, we examined feasibility of our mHealth approach and perceptions of older patients using technology so that our findings could be used in future research. An initial limitation is that we did not collect demographic data on the potential participants who did not respond to our invitation to participate. Our sample size was small; thus, we described trends in findings, reported frequencies, and did not have the power to determine significance in group outcomes. We augmented our survey data with interviews from 21 participants to help explain and understand the data, partially mitigating some limitations. This study was performed in 1 state with 2 geographically diverse health clinics and limits generalizability. We hope to have furnished valuable insights for future study.

### Conclusions

We examined the feasibility of using mHealth and automated digital equipment in older adults with HF who mostly resided in rural areas. Most participants described ease of use and satisfaction with the equipment, and problems with the equipment were essentially the same with the connected mHealth and the home equipment. We learned how older adults engage and communicate with their providers in hopes of augmenting their communication and engagement with mHealth technology in future studies. Many had problems with access to care and relied heavily on the ED for access. Our call center was acceptable to participants and their health care providers and a feasible method of promoting self-management. We found that these older adults tended to increase knowledge and skills related to self-care and could and would self-monitor for HF indices that predict decompensation and pending crises. mHealth equipment is feasible with potential to improve patient-centered outcomes and improve self-management in older adults with HF.

## Abbreviations

BP: blood pressure
ED: emergency department
HF: heart failure
mHealth: mobile health
SoC: standard of care
